# Balancing Practicality and Validity of Elder Abuse Measures: Data From Adult Protective Services Investigations

**DOI:** 10.1093/geroni/igaa057.2321

**Published:** 2020-12-16

**Authors:** Kendon Conrad, Pi-Ju Liu, Zachary Hass, Karen Conrad

**Affiliations:** 1 University of Illinois at Chicago, Chicago, Illinois, United States; 2 Purdue University, West Lafayette, Indiana, United States; 3 University of Illinos at Chicago, Chicago, Illinois, United States

## Abstract

Research in Adult Protective Services suffers from the problems of small sample size, low completion rates, and poor data quality. To address the “small sample size” issue, data were generated on 1,472 elder abuse cases over six months in California using the Identification, Services and Outcomes (ISO) Matrix. The problem of poor data quality was addressed by testing psychometric properties of Short-Forms on each type of abuse. Good reliability and predictive validity were found for all measures except those that were very rare. Finally, even shorter Mini-Forms were developed and tested in order to contribute to improved completion rates. Mini-Form results were mixed, and some will require further research on their reliability and validity, as well as examination of their ability to improve completion rates. Ongoing adoption by two California counties and the State of Montana demonstrate sustainability and increasing sample sizes using the ISO Matrix for research and practice.

